# Supplementary material for: Stigma and social support and their impact on quality of life and self-esteem among women with endometriosis in Latin-America and the Caribbean
Source: PLOS Glob Public Health. 2022 Dec 2;2(12):e0001329. doi: 10.1371/journal.pgph.0001329 (PMC10022163; doi:10.1371/journal.pgph.0001329)
Supplement: Attachment — Submitted filename: RESPONSE LETTER_Aug_25.pdf [file pgph.0001329.s002.pdf]

August 23, 2022

Dr. Rakesh Singh
Academic Editor
PLOS Global Public Health

Dear Dr. Nowrouzi-Kia,

Thanks for the opportunity to submit a revised draft of the manuscript titled "*Stigma and social support and their impact on quality of life and self-esteem among women with Endometriosis in Latin-America and the Caribbean*" to PLOS Global Public Health. We appreciate the time and effort dedicated to providing valuable feedback on the manuscript. We have incorporated all the reviewers' comments. We have highlighted all changes within the manuscript using track changes.

Below is a point-by-point response to the reviewer's comments and concerns.

For additional information, you can contact the corresponding author by email at elrivera@psm.edu.

On behalf of all authors,

Sincerely,

Eliut Rivera-Segarra, PhD
Associate Professor
School of Behavioral and Brain Sciences
Ponce Health Sciences University
Puerto Rico

RESPONSE TO REVIEWERS

We thank the reviewers for providing their comments on our manuscript. We believe they have substantially improved our manuscript. We have been able to incorporate changes to reflect most of the suggestions provided by the reviewers. We have highlighted the changes within the manuscript.

Here is a point-by-point response to the editor and reviewers' comments and concerns in the manuscript by section.

JOURNAL REQUIREMENTS:

1. Please include a complete copy of PLOS' questionnaire on inclusivity in global research in your revised manuscript. Our policy for research in this area aims to improve transparency in the reporting of research performed outside of researchers' own country or community. The policy applies to researchers who have travelled to a different country to conduct research, research with Indigenous populations or their lands, and research on cultural artefacts. The questionnaire can also be requested at the journal's discretion for any other submissions, even if these conditions are not met. Please find more information on the policy and a link to download a blank copy of the questionnaire here: https://journals.plos.org/globalpublichealth/s/best-practices-in-research-reporting. Please upload a completed version of your questionnaire as Supporting Information when you resubmit your manuscript.

Response: We have now included a complete copy of PLOS questionnaire on inclusivity in global research.

2. Please amend your detailed Financial Disclosure statement. This is published with the article. It must therefore be completed in full sentences and contain the exact wording you wish to be published.

a. State the initials, alongside each funding source, of each author to receive each grant.

b. State what role the funders took in the study. If the funders had no role in your study, please state: "The funders had no role in study design, data collection and analysis, decision to publish, or preparation of the manuscript."

Response: We have now addressed the editor's concern (See page 22 line 409-410): "The funders had no role in study design, data collection and analysis, decision to publish, or preparation of the manuscript"

3. Please send a completed 'Competing Interests' statement, including any COIs declared by your co-authors. If you have no competing interests to declare, please state "The authors have declared that no competing interests exist". Otherwise please declare all competing interests beginning with the statement "I have read the journal's policy and the authors of this manuscript have the following competing interests:"

Response: We have now addressed this concern by adding: "The authors have declared that no competing interests exist."

4. Please provide separate figure files in .tif or .eps format and removed from the manuscript file.

For more information about figure files please see our guidelines:
https://journals.plos.org/globalpublichealth/s/figures
https://journals.plos.org/globalpublichealth/s/figures#loc-file-requirements
Response: We have now removed figures from the manuscript file and included them in .tif format in a separate file.

5. In the online submission form, you indicated that "The manuscript presents the data used in this study. Dataset requests will be evaluated by the research team and shared upon reasonable request.". All PLOS journals now require all data underlying the findings described in their manuscript to be freely available to other researchers, either 1. In a public repository, 2. Within the manuscript itself, or 3. Uploaded as supplementary information.
This policy applies to all data except where public deposition would breach compliance with the protocol approved by your research ethics board. If your data cannot be made publicly available for ethical or legal reasons (e.g., public availability would compromise patient privacy), please explain your reasons by return email and your exemption request will be escalated to the editor for approval. Your exemption request will be handled independently and will not hold up the peer review process, but will need to be resolved should your manuscript be accepted for publication. One of the Editorial team will then be in touch if there are any issues.
Response: We have now addressed the editor's concern in page 1 line 17: "The manuscript presents the data used in this study. Although data is not publicly available in order to comply with the Institutional Review Board, dataset requests will be evaluated by the research team and shared upon reasonable request."

Please review your reference list to ensure that it is complete and correct. If you have cited papers that have been retracted, please include the rationale for doing so in the manuscript text, or remove these references and replace them with relevant current references. Any changes to the reference list should be mentioned in the rebuttal letter that accompanies your revised manuscript. If you need to cite a retracted article, indicate the article's retracted status in the References list and also include a citation and full reference for the retraction notice.
Response: We have now reviewed the reference and to the best of our knowledge they are in compliance with this comment.

REVIEWERS' COMMENTS:

Reviewer #1:

The article addresses an understudied health condition that is disabling physically and psychologically.Impact on many women worldwide. The article focuses on understanding the role of disease-related stigma on the quality of life among people affected by a chronic condition, which is the relevant angle that could inform policies and improve the quality of life of people affected by endometriosis. Despite the limitations, the study provides some helpful information for future studies.

1. Taking this into account, I recommend that the authors review the article and provide more information regarding the adaptation process of the questionnaires (beyond the statistical analysis). For example, it is unclear if the questionnaires were translated to Spanish, and if that was the case, how was it done.

Response: We have now addressed the reviewer's concern (See pages 8-10) adding:

1. **"The SSCI was translated to Spanish by two native Spanish-speaking authors (YM, AS). A third translator (ERS) compared the translated version with the original version and search for inconsistencies. After this process, the translation was considered final when no differences or inconsistencies were found". (See page 8, lines 157-162)**
2. **"The questionnaire was translated to Spanish using the same strategy described above." (See page 8, lines 173-174)**
3. **"This questionnaire was translated and validated for Spanish speaking populations in primary care settings". (See page 8, lines 179-180)**
4. **"…and has been adapted for Spanish speaking populations in a sample of Puerto Rican women living with endometriosis". (See page 9, lines 200-201)**
5. **"We administered the Spanish version available in the literature adapted and validated for Spanish speaking populations" (See page 10, lines 211-212)**

2. On the sociodemographic data, it will be essential to know the rationale behind the inclusion of the variables (for example, why religion was asked) as well as the exclusion of other variables such as socioeconomic status or if the informants were part of an association/support group, as well as employment status.

Response: We have addressed the reviewer's concern and decided to remove the variable of religion in page 6-7, Table 1. In the sociodemographic questionnaire, we did not ask participants if they were part of an association or support group. However, the study was disseminated via social media of different endometriosis advocacy associations.

3. Besides, while the authors recognised that they had not included health interventions among the variables analysed, a brief description of global, regional, and country-specific health programs/interventions/efforts to provide care to women living with endometriosis could allow to understand the study context.

Response: We have addressed the reviewer's concern in page 3, lines 85-88, adding: "In Puerto Rico, the diagnosis and treatment of endometriosis is often provided by community-based general gynecologists who do not always have specialized training in endometriosis. Globally, other countries and regions of the world also lack of comprehensive, multilevel interventions and integrated care for endometriosis patients."

Reviewer #2:

Thank you for an interesting and necessary paper on an understudied condition in this region. I found a few typos in the abstract. The abstract also lists endo-stigma without having explained that term previously - please write the full term and then the abbreviation.

Response: We have now addressed the reviewer's concern in page 2 and added in line 30: "The purpose of this study was to examine the mediating role of endometriosis related stigma (endo-stigma) and the moderating role of social support on the endometriosis QoL and self-esteem among women with endometriosis from Latin America and the Caribbean."
